# Effective Feedback to Improve Primary Care Prescribing Safety (EFIPPS) a pragmatic three-arm cluster randomised trial: designing the intervention (ClinicalTrials.gov registration NCT01602705)

**DOI:** 10.1186/s13012-014-0133-9

**Published:** 2014-10-11

**Authors:** Karen N Barnett, Marion Bennie, Shaun Treweek, Christopher Robertson, Dennis J Petrie, Lewis D Ritchie, Bruce Guthrie

**Affiliations:** Centre of Population Health Sciences, University of Edinburgh Medical Quad, Teviot Place, Edinburgh EH8 9AG UK; Strathclyde Institute of Pharmacy and Biomedical Sciences, University of Strathclyde, The John Arbuthnott Building, 161 Cathedral Street, Glasgow, G4 ORE UK; Health Services Research Unit, University of Aberdeen, Health Sciences Building, Foresterhill, Aberdeen AB25 2ZD UK; Department of Mathematics and Statistics, University of Strathclyde, Livingstone Tower 26 Richmond Street, Glasgow, G1 1XH UK; Centre for Health Policy, Melbourne School of Population and Global Health, The University of Melbourne, 207 Bouverie Street, Victoria, 3010 Australia; Centre of Academic Primary Care, School of Medicine and Dentistry, University of Aberdeen, Polwarth Building, Foresterhill, Aberdeen AB25 2ZD UK; Quality, Safety and Informatics Research Group, University of Dundee, Mackenzie Building, Kirsty Semple Way, Dundee, DD2 4BF UK

**Keywords:** Feedback, Medication errors, Medication review, Inappropriate medication, Randomised controlled trial, Primary health care, Family practice, Intervention development, Behaviour change, ePrescribing

## Abstract

**Background:**

High-risk prescribing in primary care is common and causes considerable harm. Feedback interventions have small/moderate effects on clinical practice, but few trials explicitly compare different forms of feedback. There is growing recognition that intervention development should be theory-informed, and that comprehensive reporting of intervention design is required by potential users of trial findings. The paper describes intervention development for the Effective Feedback to Improve Primary Care Prescribing Safety (EFIPPS) study, a pragmatic three-arm cluster randomised trial in 262 Scottish general practices.

**Methods:**

The NHS chose to implement a feedback intervention to utilise a new resource, new Prescribing Information System (newPIS). The development phase required selection of high-risk prescribing outcome measures and design of intervention components: (1) educational material (the usual care comparison), (2) feedback of practice rates of high-risk prescribing received by both intervention arms and (3) a theory-informed behaviour change component to be received by one intervention arm. Outcome measures, educational material and feedback design, were developed with a National Health Service Advisory Group. The behaviour change component was informed by the Theory of Planned Behaviour and the Health Action Process Approach. A focus group elicitation study and an email Delphi study with general practitioners (GPs) identified key attitudes and barriers of responding to the prescribing feedback. Behaviour change techniques were mapped to the psychological constructs, and the content was informed by the results of the elicitation and Delphi study.

**Results:**

Six high-risk prescribing measures were selected in a consensus process based on importance and feasibility. Educational material and feedback design were based on current NHS Scotland practice and Advisory Group recommendations. The behaviour change component was resource constrained in development, mirroring what is feasible in an NHS context. Four behaviour change interventions were developed and embedded in five quarterly rounds of feedback targeting attitudes, subjective norms, perceived behavioural control and action planning (2×).

**Conclusions:**

The paper describes a process which is feasible to use in the resource-constrained environment of NHS-led intervention development and documents the intervention to make its design and implementation explicit to potential users of the trial findings.

**Trial registration:**

ClinicalTrials.gov: NCT01602705

**Electronic supplementary material:**

The online version of this article (doi:10.1186/s13012-014-0133-9) contains supplementary material, which is available to authorized users.

## Background

Appropriate use of prescription drugs has considerable benefit to individual patients, but prescription drugs are also a major cause of avoidable harm [1-3]. Approximately 6.5% of all hospital admissions are caused by adverse drug events (ADEs), [1] and at least half of these are judged to be preventable [3]. Reducing primary care high-risk prescribing is therefore an important issue for health services. Implementing interventions have been difficult due to the complexity of defining appropriate measures of high-risk prescribing and identifying interventions that are known to be effective.

A number of measures of potentially inappropriate or high-risk prescribing have been used in research and service improvement. Examples include indicators from the Assessing Care of Vulnerable Elders (ACOVE) project, [4] the Screening Tool of Older Persons’ Potentially inappropriate Prescriptions (STOPP), and the Beers Criteria [5-7]. ACOVE and STOPP rely on manual record review and are costly to implement on a large scale. The Beers Criteria can be relatively easily measured using routine health-care data, but the drugs listed do not include those most commonly implicated in serious harm. More recently, new measures of potentially inappropriate prescribing have been defined using consensus methods [8,9] and have been shown to be both common and highly variable between practices [10]. Although not all of these prescriptions will be inappropriate, the high prevalence and variation indicates that improvement is attainable.

The PINCER trial showed that a complex intervention, combining pharmacist-facilitated improvement based on measurement of high-risk prescribing using data held in practices’ own clinical IT systems, was effective at reducing targeted prescribing [2]. Potentially, more cost effective improvement methods have been made possible by the ongoing development of centrally held patient-level prescribing datasets. NHS Scotland has implemented an ePrescribing programme which has created a Scotland-wide, patient-level prescribing data warehouse (the new Prescribing Information System (newPIS)), in which ~95% of prescribed items have a unique patient identifier attached. This makes it feasible to provide ongoing feedback on rates of high-risk prescribing to practices. If shown to be effective, providing feedback of performance with the aim of improving prescribing safety is an attractive approach because it is easily scalable and relatively inexpensive, allowing more expensive interventions to be more selective.

There is evidence that providing feedback can be effective. At the time of designing the Effective Feedback to Improve Primary Care Prescribing Safety (EFIPPS) study in 2010, the most recent Cochrane review (2006) identified 118 studies of clinical audit and feedback, although many were small and 80% were methodologically flawed or weak [11]. The median effect size was a 5% absolute improvement in binary indicators of guideline compliance, varying between studies from a 16% absolute worsening to a 70% absolute improvement. Only a very small number of studies examined feedback of safety data, mostly relating to benzodiazepine prescribing in the elderly [11]. The review identified key gaps in the literature relating to feedback, with a need for more theory-informed design [12] and ‘for head-to-head comparisons of different ways of doing audit and feedback’ [11]. This review was updated in 2012 with similar findings, concluding that there is still a need for future studies of audit and feedback to directly compare different ways of providing feedback [13].

The need for more theory-informed interventions is becoming increasingly recognised. New guidelines on behaviour change recommend the need to ‘specify the theoretical link between the intervention or programme and its outcome’ [14]. Key papers on behaviour change research have been published to help researchers apply psychological theory in developing interventions and implementing evidence-based practice [15-17].

The EFIPPS study is a joint academic and NHS initiative that aimed to develop two theory-informed formats for prescribing safety feedback, for evaluation of effectiveness (including cost) compared to simple ‘factual’ educational material in a large, cluster randomised trial [18]. This paper describes the development of the intervention which was intended to be feasible to implement at a large scale, replicated in a resource-constrained NHS context, embedded in NHS data systems from the outset and informed by both NHS priorities and behaviour change theory.

## Methods

### Trial design

This has been described in detail elsewhere, [18] but in brief, the trial is a pragmatic three-arm cluster randomised trial conducted in general medical practices in three Scottish Health Boards. General practices are randomised to one of the three arms, and the research outcomes (prescribing indicators) will be analysed at the patient level. This paper describes the initial development phase of the study including the selection of research outcomes and the development and design of each of the intervention components. The decision to implement a feedback design was driven by the intention of the NHS to use a familiar improvement method utilising the new patient-level prescribing resource, newPIS and feed back prescribing data to GP practices to enable them to monitor their own performance.

**Figure 1:**
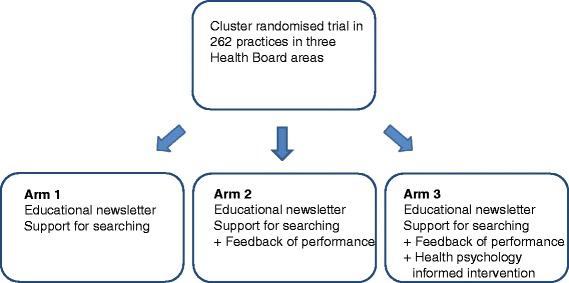
**Effective feedback to improve primary care prescribing safety trial design.**

The components delivered in each arm of the trial are shown in Figure 1. An Advisory Group was convened with representatives from the three participating Health Boards (senior GPs and pharmacists) and the technical team at NHS Scotland Information Services Division who were responsible for creating the feedback from NHS data. This NHS-led Advisory Group was responsible for selecting high-risk prescribing measures for feedback and advising on feedback design.

### Research outcomes

Outcomes had to be feasible in terms of implementation in newPIS, and consensus had to be reached among the Advisory Group as to what was important to NHS Scotland. Selection of outcomes drew on the findings of a prior RAND appropriateness method consensus study involving GPs and primary care pharmacists which identified candidate indicators [9] and evidence that the targeted prescribing was reasonably common [10]. Selection of outcomes for this trial used independent ranking of a list of candidate outcome measures by the Advisory Group members followed by in-depth face-to-face group discussion to reach consensus.

### Intervention components

This was a pragmatic trial with an overall aim of designing an intervention that could be feasibly replicated in a typically resource-constrained NHS context. As well as being plausibly effective in terms of being likely to cause a measurable change in prescribing, the intervention had to be developed over a 6-month period with relatively limited resources, deliverable within the existing IT systems at NHS Scotland Information Services Division and be acceptable to all GPs rather than a subset of research volunteers. The three intervention components included (1) educational material received by all practices (the usual care comparison), (2) feedback of practice rates of high-risk prescribing received by both intervention arms and (3) a theory-informed intervention embedded in the feedback received by only one of the intervention arms.

1. *Educational intervention and support for searching* (*same in all three arms*)*.* All educational materials were created by the research team in collaboration with the Advisory Group and where necessary topic-specific experts. Support for searching in each practice’s own IT system was also provided via the study website which made accessible to a set of downloadable searches for the general practice clinical IT systems in use in Scotland.
2. *Feedback of performance on the targeted indicators (arms two and three only).* All practices in the two treatment arms were sent prescribing feedback, with arm three additionally receiving the psychology theory-informed intervention described in the next section. Based on the data available in newPIS, the Advisory Group specified that practices should be emailed five rounds of quarterly written feedback of their rate of high-risk prescribing to mirror most prescribing initiatives that are delivered over an approximate 12-month period (and allowing for a three-month data lag in the ePrescribing Information System). Feedback was iteratively designed by the research team working with the Advisory Group. Of note is that several of the Advisory Group’s recommendations are ‘theory-informed’ in the sense that elements of behaviour change research evidence have already become ‘normal practice’. For example, several members of the Advisory Group emphasised the need for educational material to provide clear advice on what action to take and the importance of having a credible messenger in terms of who signs emails and branding of feedback. These behaviour change techniques are included in the extensive list of techniques provided by Michie and colleagues 2008 [17]. The intention was that the feedback-only arm of the trial (arm two) should represent ‘best practice’ as it would be implemented by NHS Scotland. The theory-informed intervention therefore had to implement techniques over and above those already implemented in ‘normal practice’ to allow proper assessment of the more resource intensive intervention in arm three.
3. *Theory*-*informed behaviour change intervention (arm three only).* A number of social cognition models have been developed to help predict and understand health behaviours [19]. The Theory of Planned Behaviour (TPB) is [20] a motivational model that focuses predominantly on behavioural intention provided the conceptual framework that guided the development of the psychology-informed component of the intervention. TPB is one of the more commonly used social cognition models and has been validated and used rigorously in various health settings [21,22]. While other psychological models of behaviour change could also have been considered, TPB was selected for use in the current study due to both its evidence base and the support available for health professionals to use this theory should the trial be rolled out at a national level. To predict behavioural intentions, TPB focuses on the three psychological constructs that are amenable to change: attitude, subjective norms and perceived behavioural control. A comprehensive TPB manual has been developed to help researchers construct questionnaires to measure these constructs that can then be used to inform intervention design and development [23]. While TPB is predominantly a motivational model to predict intention, perceived behavioural control is a determinant of both behavioural intention and behaviour. A meta-analysis of 87 studies looking at health behaviours found TPB accounted for 41% of the variance in intention and 34% of the variance in behaviour [21]. However, a criticism of TPB and other motivational models is the implicit assumption assuming near perfect correspondence between intention and behaviour, and studies have shown that a much larger proportion of the variance in intention is explained compared to that in behaviour [24]. To address the gap between intention and behaviour, we also drew on the Health Action Process Approach (HAPA) [25]. HAPA proposes that the process of behaviour change can be explained by two distinct phases: a motivational phase (e.g. social cognition models such as TPB) and a planning or volitional phase that focuses on action-controlled strategies. The volitional phase has three overlapping stages: planning, action and maintenance, and describes a post-intention and pre-actionable process that has a direct correlation with carrying out the action (or behaviour).

#### Target behaviours

The TPB manual highlights the importance of defining the target behaviour in specific terms, although questionnaires using TPB can also be developed to target more general behaviours [23]. The target behaviour we hoped to elicit from our intervention was that GPs would ‘respond to feedback’ by searching in their IT system for patients receiving a high-risk prescription and reviewing their prescribing. Due to the nature of the targeted behaviour, how GPs ‘respond to feedback’ could include a composite of individual behaviours that vary both by practice and for individual patients. For example, behaviours carried out by the GP might include searching for and identifying patients receiving a high-risk prescribing indicator, reviewing patient records, arranging telephone or face-to-face consultations and changing patient prescribing where appropriate. Ultimately, the decision, as to whether or not a change in medication is required, remains with the GP since some high-risk prescribing could be appropriate in the sense of the benefit outweighing the risks for a particular individual. Based on pilot work for a different trial and other published studies, [2,26] the research team and the Advisory Group strongly believed that reviewing high-risk prescribing was likely to lead to a change in medication in a significant proportion of patients. For this reason, the psychology-informed intervention has been purposefully targeted at the behaviour of ‘searching for and reviewing patients with high-risk prescriptions’ with the assumption that this behaviour will be highly correlated with a change in prescribing (the primary outcome of the trial is a composite measure of all six high-risk prescribing indicators).

#### Elicitation study (focus groups)

An elicitation study consisting of four focus groups, each comprising between six and ten health professionals was carried out to develop the measures for the three TPB psychological constructs (attitude, subjective norms and perceived behavioural control). Two focus groups were carried out with GPs: one in Tayside and one in Lothian (including members of the General Practice Prescribing Committee) and two with primary care pharmacists: one in Lothian and one in Tayside. Primary care pharmacists were invited to take part as they are often involved in initiatives to change GPs’ prescribing behaviour and could provide additional insights to those provided by GPs. Focus groups were facilitated by two members of the research team (KB, BG). The primary purpose of the focus groups was to elicit a broad range of attitudes and potential barriers to carrying out the targeted behaviour, the results of which would be used to help develop statements relating to the three TPB constructs. For example, GPs and pharmacists were asked to discuss the advantages and disadvantages of responding to the prescribing feedback, the social pressures that GPs might experience when reviewing high-risk prescribing and changing medications, the potential barriers or the facilitating factors of responding to the prescribing feedback.

#### Email Delphi questionnaire study

A two-stage email Delphi study was conducted with GPs to prioritise which questionnaire items (or statements) collated from the elicitation study were the most important (or influential) to GPs in terms of carrying out the targeted behaviour, searching for patients in their IT system and reviewing their prescribing. An invitation letter to take part in the study, including a participant information leaflet and a copy of the questionnaire, was emailed to 500 randomly selected GPs from an email address list of GPs working in Scotland (excluding GPs working in the three Health Boards taking part in the trial). GPs were asked to rate each questionnaire item, on a five-point Likert scale, their level of agreement with each statement from strongly agree to strongly disagree according to their own personal beliefs. Completed responses were received from 48 GPs (10% response rate). The 48 GPs who completed the first questionnaire were then sent the questionnaire a second time, this time including the median, minimum and maximum response scores for each statement received in round one, and a reminder of how they had personally scored each item in the first round. GPs were asked to rescore each statement using the additional knowledge of their colleagues’ collective response. In round two, 24 of the 48 GPs responded (50% response rate). Statements were deemed to be potential candidate variables if a) there was consensus of positive agreement with the importance of a statement or b) there was evidence of significant disagreement among the GP responses (>25%; if more than six GPs were outliers). Questionnaire items were excluded if there was agreement that a statement was not important in relation to reviewing patient prescribing (≥75%) or if more than 50% of GPs gave a neutral response.

The median scores from the questionnaires completed in round two were then used to identify the questionnaire items most likely to influence GPs behaviour.

#### Mapping targeted variables to behaviour change techniques

The next stage was to map the psychology constructs within each of our two models of behaviour change (TPB, HAPA) to appropriate behaviour change techniques based on the evidence as described by Michie and colleagues [17]. In this paper, Michie and colleagues generated an extensive list of behaviour change techniques and definitions from techniques published in two systematic reviews, ‘brain storming’ and a systematic search of relevant textbooks. Four experts judged which techniques would be effective at changing 11 theoretical constructs associated with behaviour change. The first step in the present study was to map the four psychological constructs: attitude, subjective norms, PBC and action planning to the corresponding constructs among the 11 identified by Michie et al. The next step was to list the techniques where the experts had reached consensus of ‘agreed use’ or were ‘uncertain’ based on the level of evidence. Techniques where there was ‘disagreement’ among the experts or ‘agreed non-use’ were not considered.

The feedback intervention in its entirety had been designed to represent ‘best practice’ and was informed by an expert Advisory Group. As a result, a number of the listed techniques were, to varying degrees, already being utilised in the feedback intervention. Examples of these included monitoring, feedback, provision of information regarding the behaviour/outcome and objects to facilitate the behaviour (e.g. support for searching in GP IT systems). The psychology-informed intervention therefore had to include techniques that were not already embedded in the other components of the feedback. This was in order that any additional effectiveness of the more resource intensive (in terms of development) theory-informed intervention could be assessed.

It is also important to note that the choice of techniques was further constrained by the nature of the trial—the theory-informed intervention had to be delivered as part of the quarterly feedback to GP practices and was limited to one A4 page (a balance between the additional time cost for GPs engaging with the intervention and encouraging behaviour change). The list of behaviour change techniques is provided in Table 1

**Table 1:** **TPB constructs and behaviour change techniques**

**Psychology construct**	**Level of evidence based on expert opinion (Michie** [17]**)**	
	**Agreed use**	**Uncertain**
**Attitude** (beliefs about consequences)	•Self-monitoring	•*Monitoring*
	•**Persuasive communication**	•Graded task
	•*Information regarding behaviour/outcome*	•**Modelling/demonstration of behaviour**
	•*Feedback*	
**Subjective norms** (social influences)	•**Social processes of encouragement, pressure, support**	•*Monitoring*
	•**Modelling/demonstration of behaviours**	**Reward/incentives**
		•Role play
		•Persuasive communication
		•Homework
**Perceived behavioural control** (beliefs about capabilities and environmental context and resources)	•Self-Monitoring	•*Monitoring*
	•**Graded task**	•**Reward/incentives**
	•**Increasing skills, problem solving, goal setting**	•Stress management
	•Coping skills	•*Information regarding behaviour*/*outcome*
	•Rehearsal of relevant skills	•Personalised message
	•**Social processes of encouragement**	
	•*Feedback*	
	•Self-talk	
	•*Environmental changes*, *e.g. objects to facilitate behaviour*	
**Action planning** (action planning)	•**Goal target specified**	•*Monitoring*
	•Contract	•Social processes of encouragement
	•**Planning/implementation**	•Personalised message
	•**Prompts, triggers, cues**	•Homework
	•Use of imagery	•*Feedback*
		•Self-talk

Marked in italics demonstrates techniques already incorporated into feedback for both intervention arms two and three.

Marked in bold demonstrates techniques selected and implemented in the psychology-informed intervention.

## Results

### Research outcomes

Six prescribing indicators were selected by the Advisory Group to be targeted by the intervention and measured at the end of the trial. The selected prescribing indicators are shown in Table 2.

**Table 2:** **Prescribing indicators selected by the Advisory Group to be used in the trial**

	**Prescribing indicators**
1.	Oral antipsychotic prescription to a patient aged 75 years and over (as a proxy of oral antipsychotic prescribing to older people with dementia).
2.	Oral non-steroidal anti-inflammatory drug (NSAID) prescription to a patient aged 65 years and over who is currently prescribed a diuretic and an ACE inhibitor or angiotensin receptor blocker (the ‘triple whammy’).
3.	Oral NSAID prescription to a patient aged 75 years and over but who is not currently prescribed a gastroprotective drug.
4.	Oral NSAID prescription to a patient aged 65 years and over who is currently prescribed either aspirin or clopidogrel but who is not currently prescribed a gastroprotective drug.
5.	Oral NSAID prescription to a patient currently prescribed an oral anticoagulant but who is not currently prescribed a gastroprotective drug.
6.	Aspirin or clopidogrel prescription to a patient currently prescribed an oral anticoagulant but who is not currently prescribed a gastroprotective drug.

### Intervention components

1. *Educational intervention* (*same in all three arms*)*.* A two-page written educational intervention was designed to be delivered to all practices by email in the month before the trial started. This mirrored existing prescribing information sent to practices in overall length and style. The Advisory Group recommended that it emphasises that high-risk prescribing is common and that it is good practice to regularly identify and review patients receiving high-risk drugs, that it clearly lists the prescribing indicators targeted, that it summarises the risk that the targeted prescribing poses and that it provides clear advice as to what actions to take. A link to an NHS Scotland hosted website with additional information for those who wish to read more is provided. [see Additional file 1: Short two-page educational material and Additional file 2: Supplementary education material available on the website].
2. *Feedback of performance on the targeted indicators* (*arms two and three only*)*.* The research team designed the feedback based on the Advisory Group specification that feedback should include (1) a clear statement of its nature (the first page has the title ‘High-risk Prescribing Feedback for Your Practice', the practice name, a statement of the purpose of feedback and a list of the indicators); (2) a graphical summary for each indicator that shows the change in their practice’s prescribing over time for each indicator, compared to a national benchmark (which was set as the lower quartile for all Scottish practices in the year before the study started, which was NHS Scotland preferred practice at the time); (3) a summary of all six indicators on a single page; (4) a detailed one-page description of each indicator, consisting of the graphical summary, a statement of why the indicator matters and what practices should do, for example, to avoid particular combinations of drugs or review patients with particular prescribing (this text is the same for all practices), and an interpretation of what the graph shows for the practice receiving the feedback (this text is automatically generated depending on the data and is unique to each practice stating, for example, the number of patients affected in the practice and a warning about sensitivity of the measure to small numbers of patients where appropriate) and (5) the emails sent should be signed by both the Health Board Medical Director and a named professional from NHS Scotland Information Services Division (ISD), and that the feedback should be branded with Health Board and ISD logos. This is also how the intervention would be implemented in practice if it was not part of a research study.
3. *Theory*-*informed behaviour change intervention (arm three only).* Two psychological models of behaviour, TPB and HAPA, were selected to inform an intervention that was designed to target the behaviour of ‘searching for and reviewing patients with high-risk prescriptions’ in general practice.

#### Elicitation study (focus groups)

The focus groups with GPs and pharmacists generated a broad range of responses regarding the advantages and disadvantages of responding to the prescribing feedback, the social pressures that GPs might experience when reviewing high-risk prescribing and changing medications, and the potential barriers/or the facilitating factors of responding to the prescribing feedback. For example, three out of the four focus groups discussed the attraction of being able to compare prescribing performance with other practices as an advantage to taking part in the intervention and reviewing patient prescribing. The fear that high-risk prescribing could lead to potential harm for the practice as well as the patient, particularly if the prescribing results in a significant event, was also thought to be an important factor that would encourage GPs to review patient prescribing. The key disadvantage that was raised in the focus group discussions was the impact on GP’s time and work load. The practice pharmacist and local GP committees were felt to have the most influence over whether or not GP practices would endorse the intervention. Patients currently doing well on their medication and carers/care workers (regarding dementia patients) were thought to the groups most likely to oppose a change in medication. The key barriers of responding to the prescribing feedback identified in the focus groups were if the instructions provided with the feedback were confusing, there was too much information or if the message was not in keeping with current advice from secondary care. The key facilitators of responding to the prescribing feedback were if GPs believed that the feedback was important and clearly branded, could be delivered face-to-face e.g. via the practice pharmacist and if the feedback was related to research and improving patient safety rather than cost-effectiveness.

#### Email Delphi questionnaire study

The broad ranges of responses obtained in the focus groups were transcribed into statements corresponding to the three TPB constructs. For example:

- Attitude—*If I respond to prescribing feedback I will feel like I am doing something positive for the patient.*
- Subjective norms**—***Approval from other GPs in my Practice is important to me.*
- Perceived behavioural control—*It would be difficult for me to respond to prescribing feedback due to limited practice resources.*

A total of 59 statements were collated and included in the Delphi questionnaire. Twenty-four GPs from various Scottish Health Boards completed both rounds of the Delphi questionnaire, scoring each statement in terms of their own beliefs of the importance or influence of the statement on the targeted behaviour: responding to feedback by searching for patients in their IT system and reviewing their prescribed medications. Consensus was reached on 50 of the 59 statements, only two statements received a neutral response where more than 50% of respondents neither agreed nor disagreed with the importance of the statement, and consensus was not met for seven of the 59 statements. A summary of the statements that reached consensus and those where there was disagreement are shown in Table 3. Neutral responses were received for two statements whether the pharmaceutical industry had an influence on a GP’s decision to review patient prescribing and whether approval from the press was important to GPs.

**Table 3:** **Summary table of the key results from the Delphi questionnaire**

**TPB constructs**	**Delphi questionnaire: results**
Attitude	*Consensus of agreement regarding ATTITUDES toward responding to prescribing feedback (≥75% agree/strongly agree; ≥6 GPs were outliers)*
	Reviewing patient prescribing was a positive thing to do for the patient.
	Reviewing patient prescribing gave GPs a sense of protecting their patients.
	The fear of a patient having a significant event as a result of receiving high-risk prescribing caused GPs concern.
	Reviewing patient prescribing was important.
	GPs do not regard receiving prescribing feedback as a criticism.
	GPs would not feel defensive in response to receiving prescribing feedback.
	*There was disagreement among GP responses to the following statements (>25%; more than 6 GPs were outliers)*
	Being seen by my colleagues to have unwittingly prescribed a high-risk drug would be embarrassing.
	A negative event as a result of changing prescribing in the past would make me less likely to change or stop medications in the future.
Subjective norms	*Consensus of agreement regarding the importance of other groups and the importance of their opinions to GPs (≥75% agree/strongly agree)*
	The groups of people most likely to approve of responding to prescribing feedback were the GMC, other GPs and practice pharmacists.
	Approval from patients and GPs within their own practice was most important, followed by approval from the GMC then the practice pharmacist.
Perceived Behavioural Control (PCB)	*Consensus of agreement regarding the BARRIERS to respond to the prescribing feedback (≥75% agree/strongly agree)*
	GPs would be less likely to respond to the prescribing feedback if clinical guidelines are unclear, or there is no sound evidence base.
	A negative event as a result of changing prescribing in the past would make GPs less likely to change or stop patient’s medication in the future.
	*There was disagreement among GP responses to the following statements (>25%)*
	I already have too much to do and would struggle to find time to review patients receiving high-risk prescribing.
	I will not respond to the prescribing feedback if it appears difficult, or it is not clear what I need to do.
	Even when it is high risk, I find it difficult to change my patients prescribing when they feel fine and are having little or no side effects from their medication.
	I find stopping medications more difficult if the medication was started in secondary care.
	Patient preferences are a key determinant for me when considering whether or not to change a patient’s medication.
	*Consensus of agreement regarding FACILITATORS of responding to the prescribing feedback (≥75% agree/strongly agree)*
	Prescribing feedback is more persuasive if the recommendations are in line with SIGN/NICE guidelines.
	The messenger is important, and GPs would be more likely to respond to the prescribing feedback that came from the practice pharmacist, a respected clinician or the Health Board.
	GPs would be more likely to respond to the prescribing feedback knowing that they would be able to benchmark the performance of their practice against other practices.
	GPs would be more likely to respond to the prescribing feedback if they could use the reviews as part of their annual appraisal.

#### Mapping targeted variables to behaviour change techniques

The research team selected behaviour change techniques from an evidence-based list, [17] for each of the four constructs within the two psychological models that had been selected to inform the intervention: attitude, subjective norms, perceived behavioural control and action planning. The Advisory Group recommended that the psychology-informed intervention should be different for each round of feedback since they believed that this would maximise the potential of it to change behaviour. It was evident from the elicitation study that the behaviour sequence that practices would engage in to ‘respond to the prescribing feedback’ would vary across practices. Of note is that the pragmatic nature of the delivery of the feedback meant that the intervention would first be sent to the practice managers’ who would then choose how to disseminate the information to the GPs. This reflects how such feedback would be implemented in the ‘real world’ but makes it harder to identify ‘specific’ behaviours to target. Results from the Delphi study confirmed that the core attitudes, social norms, barriers and facilitating factors of responding to the prescribing feedback were consistent. For this reason, the research team chose to design a behaviour change intervention for each of the four psychology constructs within the two models: attitudes (TPB), subjective norms (TPB), perceived behavioural control (TPB) and action planning (HAPA). The results from the email Delphi questionnaire were used to inform the content and design of each of the four A4 page interventions. The psychology-informed interventions are available to view as Additional file 3: Psychology-informed interventions.

Participating practices in arm three would receive interventions targeting (in order of receipt): action planning (HAPA), perceived behavioural control (TPB), attitude (TPB), subjective norms (TPB) and a repeat of the action planning intervention in the fifth round of feedback. The intervention appears as page two in the feedback document. An example of the feedback document is available to view as Additional file 4: Example feedback intervention.

## Discussion

The aim of this paper was to describe in detail the design and development of a feedback intervention for a cluster randomised trial evaluating the impact on high-risk prescribing of two different designs of feedback compared to a simple educational message. The results of the previous clinical audit feedback trials have been mixed [11], and in terms of evaluation, a major criticism has been that most have not been informed by theory or been explicit in their approach [12].

This study provides an unusual opportunity to provide a practical demonstration of how one might use theory to inform an intervention, given real-life constraints, and rigorously test the intervention in a health-care setting. This was a pragmatic trial, which was intended to be feasible to implement at a large scale by virtue of being embedded in NHS data systems from the outset, and was therefore limited by time, cost and practical constraints. While we acknowledge that a more sophisticated and costly psychology-informed intervention might plausibly produce a larger effect size, we sought to test whether investing in a ‘real-world’ feasible resource, to develop a theory-informed intervention, improved prescribing more than the development of a current ‘best practice’ form of feedback. Drawing on the behaviour change literature, we selected evidence-based theories to inform our intervention for changing prescribing behaviour. The development of our elicitation study and Delphi questionnaire was informed by TPB where we referred to the TPB manual, designed specifically to help health services researchers, use behaviour change theory to design and develop questionnaires, the results of which can then be used to help inform targeted interventions. In terms of reporting, we have been guided by the Consolidated Standards of Reporting Trials (CONSORT) group [27] and the recommendations of Michie et al. [28] and the recent introduction of the Workgroup for Intervention Development and Evaluation Research (WIDER) [29].

## Conclusions

A criticism of previous published behaviour change intervention studies has been that they have lacked explicit detail in their approach and therefore hinder their reproducibility [30]. Our aims in this paper were to describe in detail the design and development of our feedback intervention and to provide a pragmatic ‘real-life’ example of implementing a theory-informed intervention. We believe that documenting the intervention in detail is important in supporting the interpretation of the findings of the main trial and will make a useful contribution to the literature on intervention development, particularly for researchers or health service improvement professionals working within the constraints of a real-life health-care setting.

## Additional files

Additional file 1:
**Short two-page educational material.** This file contains the education material that was delivered to all three arms of the trial. Additional file 2:
**Supplementary educational material available on the website.** This file contains the supplementary educational information that was available to all three arms of the trial via the website. Practices were directed to the website, and the supplementary information, in the short educational material that was delivered to all practices. Additional file 3:
**Psychology-informed interventions.** This file contains each of the four one-page psychology informed interventions that were embedded in each of the five quarterly rounds of feedback. Action planning was repeated in the fifth round of feedback. Additional file 4:
**Example feedback intervention (arm three).** This file contains an example of one round of quarterly feedback delivered to a practice in arm three of the trial.
