# Supplementary material for: Effective Feedback to Improve Primary Care Prescribing Safety (EFIPPS) a pragmatic three-arm cluster randomised trial: designing the intervention (ClinicalTrials.gov registration NCT01602705)
Source: Implement Sci. 2014 Oct 11;9:133. doi: 10.1186/s13012-014-0133-9 (PMC4201916; doi:10.1186/s13012-014-0133-9)
Supplement: Additional file 1: — Short two-page educational material. This file contains the education material that was delivered to all three arms of the trial. [file 13012_2014_133_MOESM1_ESM.pdf]

High risk prescribing in General Practice

Prescribing is inherently risky, and clinicians and patients often have to balance benefit and harm in people with multiple and complex diseases. Patients therefore often receive prescriptions which are high-risk, but many of these prescriptions are appropriate because they are the least bad option. However, high risk prescribing needs to be regularly reviewed to ensure that the original indication still holds, and that the balance of risk and benefit hasn't changed. Below is information on 6 types of high-risk prescription which we know are commonly found in Scottish general practices. More information about each of the indicators is available at www.isdscotland.org/efipps11. We encourage you to:

- Search for patients with this type of prescribing using the EMIS and Vision searches available to download at www.isdscotland.org/efipps12.
- Review the records of patients identified, and decide if they should stop the high-risk drug or need a face to face review and discussion of benefits and risks.
- Stop the high risk prescription if possible. If not possible, then consider mitigating the risk of gastrointestinal bleeding from non-steroidal anti-inflammatory drugs by co-prescribing a proton pump inhibitor according to local guidelines.

High risk prescribing	What is the risk?	Advice	Comment
Patient aged ≥75 years, who is prescribed an antipsychotic drug (as a proxy for antipsychotic use in dementia)	Oral antipsychotics have only small effects on behavioural disturbance in dementia, but cause significant harm, causing ~1600 non-fatal strokes in, and killing ~1800 people with dementia every year in the UK.[1]	Antipsychotics **should be avoided** in older people with dementia. People with dementia prescribed an antipsychotic for behavioural disturbance should be **reviewed regularly** and **prescribing stopped** wherever possible.[1, 7]	Antipsychotics are often started for short-term behavioural disturbance at the time of hospital or care home admission, but then continued long-term. Phased withdrawal does not usually lead to significant behavioural disturbance in people with dementia with little or no behavioural disturbance while taking antipsychotics. There is more detailed advice at www.isdscotland.org/efipps11.
Patient aged ≥65 years and currently taking an ACE inhibitor/ Angiotensin Receptor Blocker and a diuretic, who is prescribed an NSAID (the 'triple whammy').	Substantially increased risk of acute renal failure and death.[2, 3]	This combination **should be avoided** particularly in those with chronic kidney disease or heart failure.[3, 7]	If NSAIDS are essential, then monitor renal function, advise patients to seek professional advice if at risk of dehydration and consider additional renal function monitoring if the patient is at risk of dehydration or unwell. The safest course of action is always to avoid the NSAID where possible.
Patient aged ≥75 years, who is prescribed an NSAID without gastroprotection.	Increases risk of gastro-intestinal bleeding 10-fold compared to NSAID use in middle age.[4, 5]	NSAIDs **should be avoided** in the elderly.6 Full-dose paracetamol or topical NSAIDs **should be tried first** for non-inflammatory musculoskeletal pain, and will provide good analgesia in many patients currently taking an NSAID.[7]	If an NSAID is essential, then use ibuprofen (≤1200mg per day) if necessary and co-prescribe a PPI like omeprazole or lansoprazole (consistent with your Board formulary). The safest course of action is always to avoid NSAIDs in the elderly where possible.

High risk prescribing	What is the risk?	Advice	Comment
Patient aged ≥65 years currently taking either aspirin or clopidogrel, who is prescribed an NSAID without gastroprotection.	Increases risk of gastro-intestinal bleeding 8-fold compared to aspirin alone.[6]	This combination **should be avoided**.[7]	If an NSAID is essential, then use ibuprofen (≤1200mg per day) if necessary and co-prescribe a PPI like omeprazole or lansoprazole (consistent with your Board formulary), but remember to prescribe a PPI other than omeprazole or esomeprazole in clopidogrel users. The safest course of action is always to avoid the NSAID where possible.
Current oral anticoagulant user prescribed an NSAID without gastroprotection.	Increases risk of gastro-intestinal bleeding by 3 to 8-fold over warfarin alone.[6]	This combination **should be avoided**.[7]	If an NSAID is essential, then use ibuprofen (<1200mg/day) if necessary, and co-prescribe a PPI like omeprazole or lansoprazole (consistent with your Board formulary). Since PPIs may increase INR, then if they are used to gastro-protect patients on warfarin, regular PPI treatment should be used even if NSAIDs are used intermittently. The safest course of action is always to avoid the NSAID where possible.
Current anticoagulant user prescribed aspirin or clopidogrel without gastroprotection.	Increases risk of gastro-intestinal bleeding 4 to 10-fold over warfarin alone.[6]	The combination of antiplatelets and warfarin **should be avoided** unless clearly recommended by a specialist, ideally with a clear statement of duration of co-prescription.[7]	If co-prescription is essential, then co-prescribe a PPI to reduce GI bleeding risk (consistent with your Board formulary), but remember to prescribe a PPI other than omeprazole or esomeprazole in clopidogrel users. There is more detailed advice at www.isdscotland.org/efipps11.

References

1. Medicines and Healthcare products Regulatory Agency and Commission on Human Medicines (2009). Antipsychotics: use in elderly people with dementia. Drug Safety Update Vol. 2 Issue 8 http://www.mhra.gov.uk/Publications/Safetyguidance/DrugSafetyUpdate/CON041211
2. Adverse Drug Reactions Advisory Committee. Beware the triple whammy! Australian Adverse Drug Reactions Bulletin 2006;25(5) http://www.tga.gov.au/hp/aadrb-0610.htm#a1
3. NHS National Prescribing Centre (2009). Important updates on drug safety from the MHRA/CHM. MeReC Monthly No. 16 http://www.npc.co.uk/merec/cardio/cdhyper/merec_monthly_no16.php
4. NHS National Prescribing Centre (2007). Cardiovascular and gastrointestinal safety of NSAIDs. MeReC Extra No. 30 http://www.npc.nhs.uk/merec/pain/musculo/merec_extra_no30.php
5. MacDonald, T. M., S. Morant, et al. (1997). Association of upper gastrointestinal toxicity of non-steroidal anti-inflammatory drugs with continued exposure: cohort study. BMJ:315;1333 http://www.bmj.com/content/315/7119/1333
6. Delaney JA, Opatrny L, Brophy JM, Suissa S (2007). Drug-drug interactions between antithrombotic medications and the risk of gastrointestinal bleeding. Canadian Med Assoc Journal;177(4):347-51 http://www.cmaj.ca/content/177/4/347
7. British National Formulary (September 2011). London: British Medical Association and The Royal Pharmaceutical Society of Great Britain.

More information is available at www.isdscotland.org/efipps11 or you can contact us with any queries at NSS.ISD-EFIPPS@nhs.net.
