# Supplementary material for: Effective Feedback to Improve Primary Care Prescribing Safety (EFIPPS) a pragmatic three-arm cluster randomised trial: designing the intervention (ClinicalTrials.gov registration NCT01602705)
Source: Implement Sci. 2014 Oct 11;9:133. doi: 10.1186/s13012-014-0133-9 (PMC4201916; doi:10.1186/s13012-014-0133-9)
Supplement: Additional file 2: — Supplementary educational material available on the website. This file contains the supplementary educational information that was available to all three arms of the trial via the website. Practices were directed to the website, and the supplementary information, in the short educational material that was delivered to all practices. [file 13012_2014_133_MOESM2_ESM.doc]

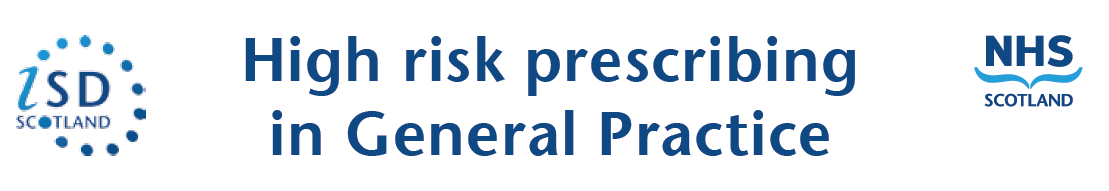


**Extended advice and references**

You can download this document from [www.isdscotland.org/efipps11](http://www.isdscotland.org/efipps11) where there are some additional documents available and downloadable searches to help you identify patients in your own practice. If you have any queries, please contact us at [NSS.ISD-EFIPPS@nhs.net](mailto:NSS.ISD-EFIPPS@nhs.net)

**Contents**

[a) Introduction 2](#__RefHeading___Toc322441929)

[b) Why review this prescribing? 2](#__RefHeading___Toc322441930)

[c) What should we do about this prescribing? 3](#__RefHeading___Toc322441931)

[d) Information about the six individual measures 4](#__RefHeading___Toc322441932)

[1. Older person (>=75 years) prescribed an antipsychotic drug 4](#__RefHeading___Toc322441933)

[2. Older person (>=65 years) currently taking an ACE inhibitor/Angiotensin Receptor Blocker and a diuretic, who is prescribed an NSAID (the 'triple whammy') 4](#__RefHeading___Toc322441934)

[3. Older person (>=75 years) prescribed an NSAID without gastroprotection 5](#__RefHeading___Toc322441935)

[4. Older person (>=65 years) currently taking either aspirin or clopidogrel who is prescribed an NSAID without gastroprotection 5](#__RefHeading___Toc322441936)

[5. Current anticoagulant user prescribed an NSAID without gastroprotection 5](#__RefHeading___Toc322441937)

[6. Current anticoagulant user prescribed aspirin or clopidogrel without gastroprotection 6](#__RefHeading___Toc322441938)

[e) When and how to reduce GI risk? 7](#__RefHeading___Toc322441939)

[References 9](#__RefHeading___Toc322441940)

## Introduction

This document contains more detailed information about the six indicators that we have sent you information about. These are:

1. Older person (>=75 years) prescribed an antipsychotic drug
2. Older person (>=65 years) currently taking an ACE inhibitor/Angiotensin Receptor Blocker and a diuretic, who is prescribed an NSAID (the 'triple whammy')
3. Older person (>=75 years) prescribed an NSAID without gastroprotection
4. Older person (>=65 years) currently taking either aspirin or clopidogrel who is prescribed an NSAID without gastroprotection
5. Current anticoagulant user prescribed an NSAID without gastroprotection
6. Current anticoagulant user prescribed aspirin or clopidogrel without gastroprotection

## Why review this prescribing?

All six indicators measure prescribing which carries considerable risk to individual patients.

Antipsychotics have been clearly shown to be only modestly effective in the management of behavioural disturbance in people with dementia. However, both traditional (e.g. haloperidol) and atypical antipsychotics (e.g. risperidone) are associated with a significantly increased risk of stroke and death, as well as with over-sedation and worsening cognition.

Numerous studies show that non-steroidal anti-inflammatory drugs (NSAIDs) and antiplatelet agents, such as aspirin and clopidogrel, are the leading causes of preventable drug related hospital admissions. Adverse events associated with antiplatelets and NSAIDs account for approximately a third of all preventable drug-related emergency hospitalisations and approximately 1% of all emergency hospital admissions.[^1^](#_ENREF_1) Additionally, antipsychotics in dementia, NSAIDs and antiplatelets are the cause of the majority of preventable drug related fatalities.

The prescribing we’re targeting will not always be inappropriate. It will sometimes be the least bad option for a particular individual and only the caring clinician is in a position to make a judgement about appropriateness. Nevertheless, it is clearly high-risk. We therefore encourage you to review patients with this prescribing on a regular basis to ensure appropriateness and minimise risk and harm.

## What should we do about this prescribing?

The key actions are to be alert to risk factors (such as age, or co-prescription) that put patients at high risk of adverse drug effects and to avoid starting drugs in these patients if possible. When drugs are considered to be essential despite being high-risk, it is crucial to ensure regular review that goes beyond the routine annual ‘medication review’ for QOF.

Vision or EMIS searches to identify patients at risk are available at [www.isdscotland.org/efipps12](http://www.isdscotland.org/efipps12). Since only you and the patient can decide what is appropriate, then what you choose to prescribe is entirely up to you, but options include:

**When considering starting NSAIDs and antipsychotics:**

- Look for risk factors that put patients at high risk of adverse events and avoid starting antipsychotics and NSAIDs in these patients.

**When reviewing older people with dementia taking antipsychotics:**

- If the patient **DOES NOT** have significant behavioural disturbance while taking it, then stop the antipsychotic, in a staged reduction if necessary (see p4). There is no need to substitute an alternative unless their behaviour or situation changes.
- If the patient **DOES** have significant behavioural disturbance while taking it, then consider and treat other causes of disturbance (such as pain), and/or specialist referral to review antipsychotic use and possible alternatives.
- Alzheimer’s Society have best practice guidance on managing behavioural disturbance in people with dementia which includes advice on starting and stopping antipsychotics <http://www.alzheimers.org.uk/site/scripts/download_info.php?fileID=1163>

**When reviewing high-risk patients taking NSAIDs:**

- Stop the NSAID, which will often mean trying another analgesic. In many older people with osteoarthritis or minor musculoskeletal injury, regular or full dose paracetamol (6-8 500mg tablets a day) is as effective as an NSAID without the risk, and patients with localised problems may find topical NSAIDs provide good analgesia without any significant risk. Patients have often not tried taking *regular* paracetamol before consulting.
- Stop the antiplatelet drug, because it is no longer appropriate (for example, long term dual use of aspirin and clopidogrel when the clopidogrel was only intended to be short term after a heart attack) or because the benefits may not outweigh the risks in this particular patient (for example, if aspirin is being used for primary prevention).
- Add gastro-protection if the risk relates to gastrointestinal bleeding. Note that this reduces but does not abolish the excess risk of bleeding. Detailed information about gastro-protection is below.
- Stop another drug that contributes to the risk if there is no strong indication for it (eg a diuretic or an ACEI/ARB enhancing the risk of renal adverse effects, or warfarin increasing the risk of and severity of bleeding).
- Agree with the patient or family that the benefit of the antipsychotic or NSAID in them is worth the risk, for example because alternatives have been tried and were not effective or had intolerable side effects.

## Information about the six individual measures

### Older people (>=75 years) prescribed an antipsychotic drug

*How high is the risk?* It is estimated that treating 100 people with dementia and behavioural disturbances with an atypical antipsychotic drug for around 12 weeks would lead to:[^2^](#_ENREF_2)

- 9 to 20 patients with at least some symptom improvement (although many improve anyway)
- 1 additional death
- 2 additional cerebrovascular adverse events (primarily stroke), of which one will be severe
- 6 to 9 additional patients with gait disturbances
- 3 to 6 additional patients with urinary tract symptoms
- A significant number of patients with sedation, fatigue or cognitive decline

It is important to note that this balance of risks and benefits might change markedly with longer periods of treatment. There is a small immediate benefit in terms of reduced behavioural disturbance (although in most patients it is not clinically significant) that is unlikely to get larger over time. However, there is evidence that adverse events continue to accumulate (at least in terms of mortality).[^2^](#_ENREF_2) Based on the DART-AD trial which randomised patients with dementia in nursing homes to continue antipsychotics or stop them,[^3^](#_ENREF_3) it is estimated that continuation of antipsychotic medication compared to cessation causes an additional 5 deaths at 12 months per 100 patients continuing treatment, and an additional 20 deaths at 24 months.

*Current guidance:* NICE guidance states that “people with Alzheimer’s disease, vascular dementia or mixed dementias with mild-to-moderate non-cognitive symptoms should not be prescribed antipsychotic drugs because of the possible increased risk of cerebrovascular adverse events and death.”[^4^](#_ENREF_4) Where prescription is unavoidable, then “treatment should be time limited and regularly reviewed (every 3 months or according to clinical need).”[^4^](#_ENREF_4) Always consider and if appropriate treat other causes of behavioural disturbance, including pain.

*Stopping antipsychotics safely:* Patients who **DO NOT** have significant behavioural disturbance while taking antipsychotics, are least likely to have any adverse events from stopping. Patients who **DO** have severe disturbance while taking antipsychotics, or in whom a previous attempt to stop led to significant worsening of symptoms, are at much higher risk of developing troublesome symptoms from stopping. Patients on low dose antipsychotics can simply have them stopped. Examples of low doses are risperidone 0.5mg daily, olanzapine 2.5mg daily, quetiapine 50mg daily, aripiprazole 5mg. Patients on larger doses can have them tapered over 4 weeks, for example an initial halving of the dose before cessation.[^5^](#_ENREF_5) More details including treatment algorithms are available on the EFIPPS educational material web pages [www.isdscotland.org/efipps11](http://www.isdscotland.org/efipps11)

*If the antipsychotic is judged to be essential:* The only licenced indication is the use of risperidone for short-term (<6 weeks) use in persistent severe aggression in Alzheimer’s type dementia that has not responded to non-pharmacological approaches and where there is a clear risk of harm to the patient or others.[^6^](#_ENREF_6) The lowest effective dose should be used for the shortest possible duration, and regular review of continued need to prescribe is required.

### Older person (>=65 years) currently taking an ACE inhibitor/Angiotensin Receptor Blocker and a diuretic, who is prescribed an NSAID (the 'triple whammy')

*How high is the risk?* Recent articles have coined the term ‘triple whammy’ to emphasise the high renal risk associated with patients prescribed all three of NSAIDs (including COXII selective agents), diuretics and ACEIs or ARBs.[^7^](#_ENREF_7) The fatality rate for cases of acute renal failure with the "triple whammy" may be as high as 10%.[^8^](#_ENREF_8)

*Current guidance:* The combination should be avoided in all patients, but the risk is likely to be higher in elderly patients and particularly those with pre-existing renal impairment or with chronic heart failure.[^9^](#_ENREF_9)

*If the NSAID is judged to be essential:* The lowest effective dose should be used for the shortest possible duration. All patients will require close monitoring of renal function and should be advised to seek medical advice if they encounter conditions causing dehydration such as diarrhoea or vomiting, since dehydration may precipitate acute renal failure in these patients.[^10^](#_ENREF_10)

### Older person (>=75 years) prescribed an NSAID without gastroprotection

*How high is the risk?* NSAIDs increase the risk of gastrointestinal bleeding at all ages, but people aged >65 years have 5 to 6 times the risk of younger people, and those aged >75 years have 10 times the risk.[^11^](#_ENREF_11) The BNF states that “NSAIDs should be used with caution in the elderly (risk of serious side-effects and fatalities)” and that “Bleeding associated with aspirin and other NSAIDs is more common in the elderly who are more likely to have a fatal or serious outcome. NSAIDs are also a special hazard in patients with cardiac disease or renal impairment which may again place older patients at particular risk.”[^12^](#_ENREF_12)

*Current guidance:* In the absence of clear inflammation, NSAIDs have no clear analgesic benefit over other drugs, although individual response to painkillers varies. NICE advises that for osteoarthritis and minor musculoskeletal injury in the elderly, full dose paracetamol and/or topical NSAIDs should be tried first and will be effective in the majority of patients.[^13^](#_ENREF_13)

*If the NSAID is considered to be essential:* If other analgesics have been tried and oral NSAIDs/COX-2 inhibitors are judged necessary, these agents should be used at the lowest effective dose for the shortest possible period of time. Standard-dose ibuprofen (≤1200mg per day) is recommended as an appropriate first choice NSAID in view of its relatively lower risk of gastrointestinal (GI) and cardiovascular (CV) side effects.[^14^](#_ENREF_14) Naproxen may be considered in patients, who are also at increased cardiovascular risk.

⮊ *Please also see section e) ‘When and how to reduce GI risk?’*

### Older person (>=65 years) currently taking either aspirin or clopidogrel who is prescribed an NSAID without gastroprotection

*How high is the risk?* Aspirin alone approximately doubles the risk of GI bleeding and co-prescribing an NSAID increases that risk to approximately 8-fold compared to untreated patients.[^15^](#_ENREF_15)

*Current guidance:* The Committee for the Safety of Medicines (CSM) has advised that co-prescription of low dose aspirin and NSAIDs should only be used if absolutely necessary.[^16^](#_ENREF_16) The BNF advises that “the combination of a NSAID and low-dose aspirin can increase the risk of gastro-intestinal side-effects; this combination should be used only if absolutely necessary and the patient should be monitored closely.”[^12^](#_ENREF_12) Co-prescription with clopidogrel is also associated with significantly increased risk.

*If combined treatment with low dose aspirin and NSAIDs is considered to be essential:* The use of PPIs as a strategy to mitigate GI risk is superior to replacing traditional NSAIDs by Cox2 selective agents (without gastro-protection), because low dose aspirin negates the gastro-protective effect of Cox-2 selective NSAIDs. [^17^](#_ENREF_17) In addition, ibuprofen and naproxen (but not diclofenac) have a more favourable cardiovascular risk profile than Cox-2 selective NSAIDs and therefore have advantages in patients using low dose aspirin for cardio-protection.[^18^](#_ENREF_18)

⮊ *Please also see section e) ‘When and how to reduce GI risk?’*

### Current anticoagulant user prescribed an NSAID without gastroprotection

*How high is the risk?* Warfarin alone increases the risk of gastro-intestinal bleeding approximately two-fold and co-prescription of an NSAID increases that risk to 3 to 8-fold compared to untreated patients.[^19^](#_ENREF_19)

*Current guidance:* The BNF states that the combination of warfarin and NSAIDs should be avoided.[^12^](#_ENREF_12)

*When prescribing PPIs to patients on warfarin:* Omeprazole and other proton pump inhibitors have the potential to enhance the effect of warfarin on the INR.[^16^](#_ENREF_16) Intensified monitoring is therefore recommended during initiation of PPIs in patients on stabilised warfarin treatment. If the decision to use gastro-protection is made in patients who use NSAIDs only intermittently, then regular PPI treatment has advantages in order to avoid INR fluctuations due to intermittent PPI use.

⮊ *Please also see section e) ‘When and how to reduce GI risk?’*

### Current anticoagulant user prescribed aspirin or clopidogrel without gastroprotection

*How high is the risk?* Warfarin alone increases the risk of gastro-intestinal bleeding approximately two-fold and co-prescription of low dose aspirin increases that risk to 4 to 10-fold compared to untreated patients.[^19^](#_ENREF_19)

*Current guidance:* The BNF states that the combination of low dose aspirin and warfarin should be avoided if possible.[^16^](#_ENREF_16) Current guidance by the European Society of Cardiology discourages combining clopidogrel and warfarin unless absolutely necessary.[^20^](#_ENREF_20)

*If dual anticoagulation and antiplatelet therapy is considered to be essential:* Dual treatment with antiplatelets and anticoagulants is indicated only for the management of patients with prosthetic heart valves, for those with peripheral arterial disease and failing grafts, and in patients with atrial fibrillation after acute coronary syndrome including myocardial infarction (for up to 12 months). In these patients, concomitant use of PPIs is usually recommended.[^22^](#_ENREF_22) As with dual use of aspirin and clopidogrel, it is important to regularly review that both drugs are required, since guidelines may change. It is likely that your practice will have some patients with stable ischaemic heart disease in whom a decision to use dual aspirin/warfarin treatment was made in the past, before current guidelines were published. Continued dual treatment in these patients will often not be indicated, and you may wish to either stop the aspirin, or to seek specialist advice by letter about discontinuing if the indication is ambiguous.

⮊ *Please also see section e) ‘When and how to reduce GI risk?’*

## When and how to reduce GI risk?

#### When to use gastroprotection in NSAID users?

- Gastro-protective drugs significantly reduce the bleeding risk of NSAIDs and antiplatelets, but they do not abolish the excess risk. The safest course of action is therefore always to stop the NSAID or antiplatelet unless there is a clear indication for it. If not, then gastro-protection is indicated in high risk patients.
- Gastro-intestinal damage can develop early (<1 month) in NSAID therapy and the risk of GI complications remains constant over the course of treatment.[^23^](#_ENREF_23) That means that although long-term treatment carries a greater cumulative risk of bleeding, even people with one-off prescriptions are at increased risk. Symptoms are a poor guide to bleeding risk because about half of upper gastro-intestinal bleeds occur *without* previous symptoms of dyspepsia. Even if the intended duration of NSAID treatment is short, patients at increased risk of developing GI bleeding should therefore promptly receive adequate gastro-protection, commencing at the same time as NSAID therapy.[^24^](#_ENREF_24) The safest course of action is to use another analgesic wherever possible.

#### Which gastro-protective drug at which dose?

- Proton pump inhibitors (PPIs) and full dose misoprostol (800mcg/d) are superior to H_2_ antagonists in the prevention of GI complications in high risk patients, even at doubled H_2_ antagonist dose (e.g. ranitidine 300mg twice daily).
- PPIs are superior to misoprostol in preventing duodenal ulcers and have a more favourable profile of undesirable side effects.[^24^](#_ENREF_24) Low dose misoprostol (e.g. 200mcg in Arthrotec or Napratec) is less effective than PPIs in preventing gastrointestinal complications and has more side effects. Combination preparations of NSAIDs with low dose misoprostol are therefore *not* currently recommended to prevent GI complications of NSAIDs.[^13^](#_ENREF_13)
- The MHRA advises that omeprazole and esomeprazole should be avoided in patients taking clopidogrel.[^27^](#_ENREF_27) In light of recent evidence, the [previous MHRA advice](http://www.mhra.gov.uk/Publications/Safetyguidance/DrugSafetyUpdate/CON051770) (to avoid all PPIs unless absolutely necessary for patients taking clopidogrel) is no longer considered necessary. Nevertheless, as a precaution, **concomitant use of clopidogrel with omeprazole or esomeprazole is discouraged.** The current evidence does not support extending this advice to other PPIs. However, because it is not possible to completely exclude a possible interaction of clopidogrel and (any) PPIs on the basis of available data, the potential risk of a slight reduction in efficacy of clopidogrel should be weighed against the potential gastrointestinal benefit of the PPI. Other gastrointestinal therapy such as double dose H2 receptor antagonists (except cimetidine), such as Ranitidine 300mg twice daily (unlicensed dose), may be more suitable in some patients.[^28^](#_ENREF_28)
- The following doses of PPIs are licensed for use in people who require continued NSAID treatment:

| **Proton Pump Inhibitor** | Dose for NSAID prophylaxis |
| --- | --- |
| Lansoprazole | 15–30 mg once daily. Higher doses of 30 mg may be more appropriate to prevent ulcer recurrence. |
| Omeprazole | 20 mg once daily |
| Esomeprazole | 20 mg once daily |
| Pantoprazole | 20 mg once daily |
| Rabeprazole sodium | Not licensed |

In patients who need to continue NSAID treatment after an ulcer has healed, full dose proton pump inhibitor therapy should usually be used.[^16^](#_ENREF_16)

#### When is Helicobacter pylori eradication indicated?

#### In patients who have *not* previously been treated with NSAIDs who now *require* an NSAID: Eradication of *H. pylori* before starting long term (>3months) treatment at least halves the overall risk that these patients will develop a peptic ulcer and is therefore recommended. [^29^](#_ENREF_29)

#### In patients who are already treated with a long-term NSAID: Eradication of *H. pylori* is unlikely to reduce the risk of peptic ulcer development in this group of patients. Maintenance PPI therapy alone is significantly more effective in preventing ulcers than HP eradication. [^29^](#_ENREF_29)

## References
