# Supplementary material for: Effective Feedback to Improve Primary Care Prescribing Safety (EFIPPS) a pragmatic three-arm cluster randomised trial: designing the intervention (ClinicalTrials.gov registration NCT01602705)
Source: Implement Sci. 2014 Oct 11;9:133. doi: 10.1186/s13012-014-0133-9 (PMC4201916; doi:10.1186/s13012-014-0133-9)
Supplement: Additional file 3: — Psychology-informed interventions. This file contains each of the four one-page psychology informed interventions that were embedded in each of the five quarterly rounds of feedback. Action planning was repeated in the fifth round of feedback. [file 13012_2014_133_MOESM3_ESM.pdf]

Please Read

Working in Primary Care we understand that GPs face considerable time pressures every day. However, due to the potential for significant harm to your patients it is important that you do review patients with the high-risk prescribing being measured. The searches you need to identify these patients within your GP system have already been developed for you to make it much easier and to minimise the time you will need to identify these patients.

Many GPs have found that making a very specific action plan is extremely useful in helping them respond efficiently to high risk prescribing feedback. An action plan outlines what has to be done, who by and when it has to be done by.

Of course, only you can develop the right action plan for you, but we have provided an example of an action plan that will help you identify and review patients with high risk prescribing.

Example Action Plan to identify and review patients receiving High Risk Prescribing

1. After surgery today, I will arrange for my practice manager to download the Vision/Emis searches from (web address).
2. I will ask him/her to run the searches tomorrow, and generate a list of the patients receiving the targeted high risk drugs to discuss in the practice meeting.
3. In the practice meeting, we will agree which doctor will review which indicator/which patients.
4. On Wednesday afternoon, I will review the notes of the patients allocated to me, and organise any follow-up necessary (record why no change is necessary in the notes; write to the patient asking them to change their medication; ask the practice manager to arrange a telephone or face to face appointment; seek clarification from a specialist).
5. I will use follow-up appointments to stop high risk prescribing wherever possible, and will complete the follow-up appointments by the end of next week.
6. I will make sure that we review progress for all indicators/patients in the practice meeting in four weeks' time.

We suggest you make a plan similar to the above that will suit you and your practice. Asking someone to monitor your progress has been shown to help successfully implement your plan. Keeping your action plan in a prominent place (and/or setting up email/phone alerts) to remind you, will ensure the process is both manageable and efficient.

Please download your action plan template at (web address).

It is a good idea to retain your action plan as this can be used as a discussion tool to demonstrate good clinical practice at your next appraisal!

Please Read

The prescribing indicators we are asking you to review are all evidence based and are focused on improving patient safety. We appreciate there are some situations or patients who present particular challenges, but there are ways of overcoming these.

Stopping antipsychotics prescribed to an older person with dementia
Mrs McPhee is 78 and has moderately severe dementia. She lives with her husband who is her main carer. A psychogeriatrician started her on quetiapine for disturbed behaviour last year, but no recommendation about treatment duration, and no follow-up. She has had no behavioural symptoms recently, but her husband is concerned about stopping the "specialists" drug.

Involve patients and carers in the decision. Research shows that stopping antipsychotics in people without behavioural disturbance is *not* associated with any worsening of symptoms, but does reduce risk of adverse events. Explaining this to patients and carers, while also accepting that it isn't possible to predict what will happen to individuals, can be helpful.
Plan for reduction and for potential problems. Agreeing a careful and gradual reduction in dose, with a clear plan if symptoms do return is also useful (permission to increase the dose again, alternative drug treatment including pain management, alternative behavioural approaches). ***Consult with specialists.*** In some situations, it will be helpful to consult with specialists, either by letter or by referral. This is definitely indicated if there are symptoms of psychosis, and may be indicated if the drug was initiated by a specialist but its indication and duration are not clear.

Stopping NSAIDs
Mr Duguid is 76 and takes diclofenac most days for mild knee osteoarthritis. He has never had side effects and is not on gastroprotection. He has mild angina and also takes aspirin. He isn't keen to change it because "it works with nae bother", although hasn't actually tried an alternatives. Last time you stopped someone's painkillers, they were upset because the alternative didn't work.

Explain the risk. Although he has never had any problems, diclofenac is risky in him because of his aspirin co-prescription, because of his age, and because it is not ideal in someone with angina. The risk will increase as he ages. Patients almost always appreciate clear and properly explained attempts to ensure that they have both *effective* and *safe* treatment.
Agree a strategy to change and plan for potential problems. The majority of patients with non-specific musculoskeletal pain will have equally good pain relief using *regular* paracetamol or topical NSAIDs if pain is localised. Intermittent use of co-codamol may also be helpful. If alternatives are clearly not adequate, then *prescribe gastroprotection* if he ends up back on the NSAID.

More information about the indicators is available at isdscotland.org/efipps31.

Reviewing these high risk prescribing indicators will improve patient safety in your practice. The work that you put into carrying out these reviews can be used as part of your next appraisal!

Please Read

From our exploratory work we know that high risk prescribing is an important topic for GPs in Scotland and they value feedback about it, because its focus is solely on patient safety and not cost effectiveness. GPs have told us that reviewing their patients prescribing makes them feel like they are doing something positive for their patients and gives them a sense of protecting their patients. Other Scottish GPs involved in this kind of prescribing safety improvement work had this to say about how responding to the feedback benefitted their patients, their fear of causing an adverse event and their embarrassment at finding that they had unknowingly prescribed a high risk drug.

"The actual reviewing [of] the patients that needed to be reviewed, we saw this as just clinical, these are people that should have been seen anyway so that was not additional work, that wasn't a burden at all."

"It was a good experience I would say. There were big improvements for the patients obviously."

Adverse events due to high risk prescribing may appear to be uncommon but when they do occur it is an extremely negative experience for all who are involved. Serious adverse events account for approximately 6.5% of all hospital admissions, half of which could and should be prevented.

"The topic is, I would go so far as to say, essential. I don't even think you can say it's [just] urgent. It's essential that practices are not doing this [high risk prescribing]. They could be killing patients totally unnecessarily and it's not as if it's difficult, because in a lot of circumstances, the vast majority of them are non-steroidals in elderly people."

"...so, I think if people find a cause and a problem and identify something [like] the triple whammy, it's potentially dangerous, potentially lethal, you could even say, that we're duty bound to do something about it..."

GPs have said that unknowingly prescribing a high risk indicator can be embarrassing. The reality is that everyone can makes mistakes, even GPs, and prescribing errors do occur. What's important is that once these errors have been identified appropriate action is taken to ensure the safety of the patient.

"...I've always thought I've been quite cautious with NSAIDs but then again possibly some of these patients were mine. You know, they've been started on NSAID despite them being on an ACE inhibitor and a diuretic and you think oh god, that's incredibly embarrassing but I think you know, I hope that I'd be extra cautious and I think I will be, particularly with NSAIDs."

We recommend that you download the searches available at (web address) and review any patients identified. More information about the indicators is available at (web address).

Reviewing these high risk prescribing indicators will improve patient safety in your practice. The work that you put into carrying out these reviews can be used as part of your next appraisal!

Please Read

Primary care prescribing can cause considerable harm with adverse drug events accounting for 6.5% of all hospital admissions. Although treatment may start in both primary and secondary care, GPs are responsible for prescribing most of the drugs that cause these admissions and for reviewing patients to ensure that their prescribing is safe.

Reviewing high risk prescribing is also important to and supported by your fellow GPs, pharmacist colleagues and of course patients. Other Scottish GPs involved in this kind of prescribing safety improvement work found taking part had benefits for their patients.

"The actual reviewing of the patients that needed to be reviewed we saw this as just clinical, these are people that should have been seen anyway so that was not additional work, it wasn't a burden at all."

"It was a good experience. There were big improvements for the patients obviously."

"These audits, reviews, call them what you like were very useful to me for identifying patients who we were, let's be polite, mistreating and we could go much stronger than that, [we were] putting at risk."

As you know, the GMC has introduced a revalidation system where you will have to renew your licence to practice every five years. One of the changes to the revalidation process will include a more rigorous annual appraisal during which you will demonstrate that you provide safe and effective care, and appropriately review the quality of your care. The high risk prescribing indicators outlined in this study are purely safety focused and not about saving money. Reviewing patients receiving these high risk indicators in your practice will provide you with evidence that can be used to demonstrate the safety of your prescribing, and that you are a reflective practitioner. Again, GPs involved in similar prescribing safety improvement work have found taking part to be both a rewarding and valuable experience.

"[Taking part] has improved prescribing practice, improved record keeping, improved our knowledge base and if people are on unsafe meds [this process has helped] getting them off them. And if they are on unsafe meds, taking part has helped us carefully consider why and ensure that it's not accidental."

"It's been good, it's been very educational and an informative [process]."

We recommend that you download the searches available at (web address) and review any patients identified. More information about the indicators is available at (web address).

Reviewing these high risk prescribing indicators will improve patient safety in your practice. The work that you put into carrying out these reviews can be used as part of your next appraisal!
