# Supplementary material for: Effective Feedback to Improve Primary Care Prescribing Safety (EFIPPS) a pragmatic three-arm cluster randomised trial: designing the intervention (ClinicalTrials.gov registration NCT01602705)
Source: Implement Sci. 2014 Oct 11;9:133. doi: 10.1186/s13012-014-0133-9 (PMC4201916; doi:10.1186/s13012-014-0133-9)
Supplement: Additional file 4: — Example feedback intervention (arm three). This file contains an example of one round of quarterly feedback delivered to a practice in arm three of the trial. [file 13012_2014_133_MOESM4_ESM.pdf]

High-Risk Prescribing Feedback For Your Practice

12345 Trial Practice, somewhere in Scotland

Report date 24 October 2013 (data complete until 30 June 2013)

This data is provided to you by NHS Scotland Information Services Division based on prescriptions dispensed to your patients for the period up until 30 June 2013. Because of changes to the way that prescriptions are processed, we are now able to provide you with new information about high-risk prescribing to individual patients. This report contains data about your practice for a set of high-risk prescribing indicators. We emphasise that 'high-risk' does not mean inappropriate, since there will sometimes be good reason for the patterns of prescribing being measured. However, these are prescriptions which do require regular review, and we encourage you to consider searching for patients with this prescribing in your own clinical IT system, to decide if they need more formal review. Advice on searching for patients in Vision and EMIS including downloadable searches can be found at www.isdscotland.org/efipps33.

Indicators included in this report:

1. Older person (>=75 years) prescribed an antipsychotic drug
2. Older person (>=65 years) currently taking an ACE inhibitor/Angiotensin Receptor Blocker and a diuretic, who is prescribed an NSAID (the 'triple whammy')
3. Older person (>=75 years) prescribed an NSAID without gastroprotection
4. Older person (>=65 years) currently taking either aspirin or clopidogrel who is prescribed an NSAID without gastroprotection
5. Current anticoagulant user prescribed an NSAID without gastroprotection
6. Current anticoagulant user prescribed aspirin or clopidogrel without gastroprotection

Further information

Please contact us if you have any queries - NSS.ISD-EFIPPS@nhs.net

Please Read

Working in Primary Care we understand that GPs face considerable time pressures every day. However, due to the potential for significant harm to your patients it is important that you do review patients with the high-risk prescribing being measured. The searches you need to identify these patients within your GP system have already been developed for you to make it much easier and to minimise the time you will need to identify these patients.

Many GPs have found that making a very specific action plan is extremely useful in helping them respond efficiently to high risk prescribing feedback. An action plan outlines what has to be done, who by and when it has to be done by.

Of course, only you can develop the right action plan for you, but we have provided an example of an action plan that will help you identify and review patients with high risk prescribing.

Example Action Plan to identify and review patients receiving High Risk Prescribing

1. After surgery today, I will arrange for my practice manager to download the Vision/Emis searches from www.isdscotland.org/efipps33.
2. I will ask him/her to run the searches tomorrow, and generate a list of the patients receiving the targeted high risk drugs to discuss in the practice meeting.
3. In the practice meeting, we will agree which doctor will review which indicator/which patients.
4. On Wednesday afternoon, I will review the notes of the patients allocated to me, and organise any follow-up necessary (record why no change is necessary in the notes; write to the patient asking them to change their medication; ask the practice manager to arrange a telephone or face to face appointment; seek clarification from a specialist).
5. I will use follow-up appointments to stop high risk prescribing wherever possible, and will complete the follow-up appointments by the end of next week.
6. I will make sure that we review progress for all indicators/patients in the practice meeting in four weeks' time.

We suggest you make a plan similar to the above that will suit you and your practice. Asking someone to monitor your progress has been shown to help successfully implement your plan. Keeping your action plan in a prominent place (and/or setting up email/phone alerts) to remind you, will ensure the process is both manageable and efficient.

Please download your action plan template at www.isdscotland.org/efipps34.

It is a good idea to retain your action plan as this can be used as a discussion tool to demonstrate good clinical practice at your next appraisal!

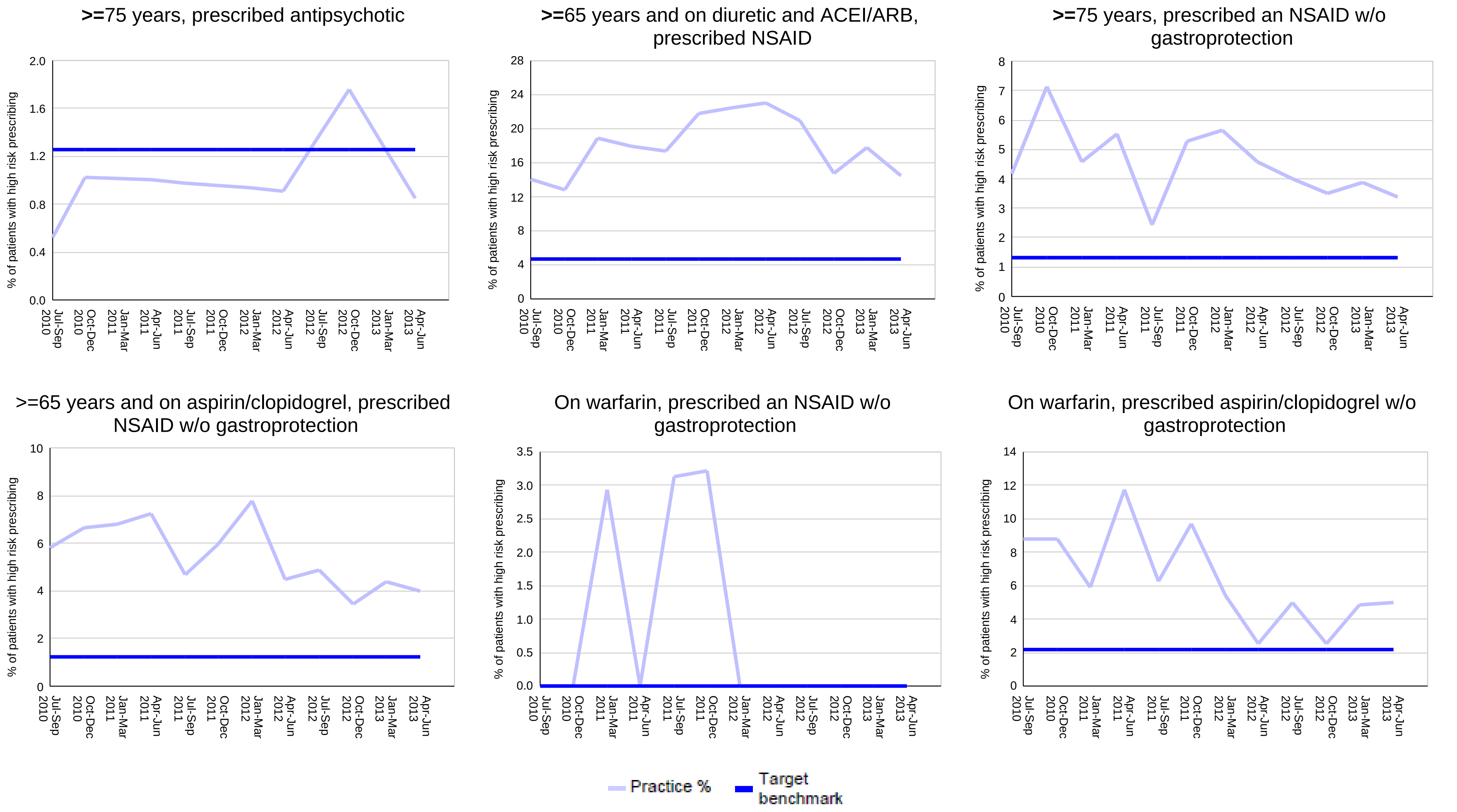

- Each chart shows changes over time the percentage of patients who are vulnerable to adverse effects of a particular group of drugs, who receive one of those drugs (for example, the percentage of patients prescribed warfarin who have also been prescribed an oral NSAID). A more detailed explanation is provided in the pages that follow.
- The light blue lines show data for your practice. The dark blue line is a benchmark showing the level which the 25% of practices with the lowest rates on this indicator achieve.

Indicator 1. Older person (>=75 years) prescribed an antipsychotic drug

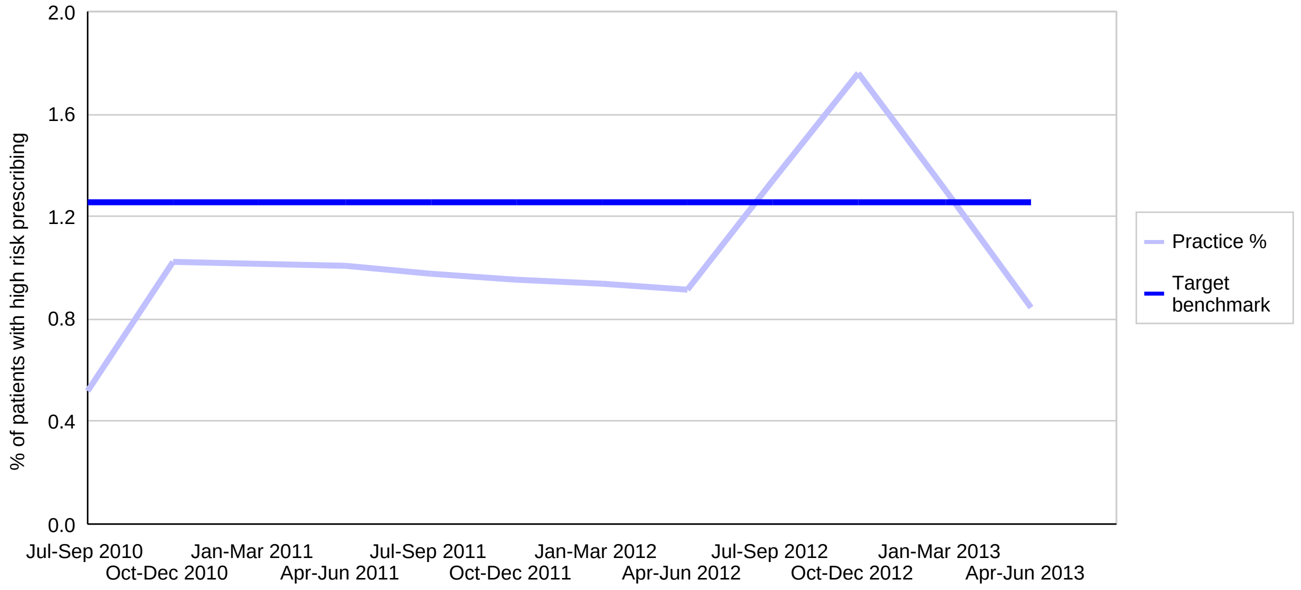

What does this indicator measure?

This indicator measures the proportion of people aged 75 and over who are prescribed an antipsychotic. This is a proxy for antipsychotic prescribing in older people with dementia, and most (but not all) patients identified will have dementia or cognitive impairment.

Why does this matter?

In older people with dementia there is good evidence that antipsychotics have only small to minimal benefits in terms of reducing behavioural disturbance. There is also good evidence that they significantly increase the risk of stroke and death and are associated with many other side effects including over-sedation and falls. It is estimated that ~1800 older people with dementia of all severities die every year in the UK as a result of antipsychotic prescribing. Current guidelines say that antipsychotics should only be used in people with dementia as a last resort for the management of severe behavioural disturbance that puts the patient or carers at significant risk of harm. At least 2/3 of antipsychotic prescribing in older people does not meet this criteria. There is trial evidence that stopping antipsychotics in people with dementia who are not behaviourally disturbed does NOT lead to significant increases in problematic behaviour. More anecdotally, antipsychotics appear to often be initiated at the time of care transitions (acute hospital admission; admission to a nursing home) which increase confusion, but that initial prescription to manage a temporary change is usually continued without full review. It is therefore recommended that all people with dementia prescribed an antipsychotic be regularly reviewed, and antipsychotics stopped wherever possible. This recommendation applies to ALL antipsychotics.

What does the information show for my practice?

You had 2 (0.85%) patients aged >=75 who were prescribed an antipsychotic in April to June 2013. This was better than the benchmark of 1.26% (the dark blue line benchmark is set at the percentage achieved by the quarter of practices with the lowest rates in financial year 2011/12). However, we would still encourage you to consider reviewing these patients since all such prescribing carries risk. Searches to identify these patients in Vision or EMIS are available at www.isdscotland.org/efipps33.

Indicator 2. Older person (>=65 years) currently taking an ACE inhibitor/Angiotensin Receptor Blocker and a diuretic, who is prescribed an NSAID (the 'triple whammy')

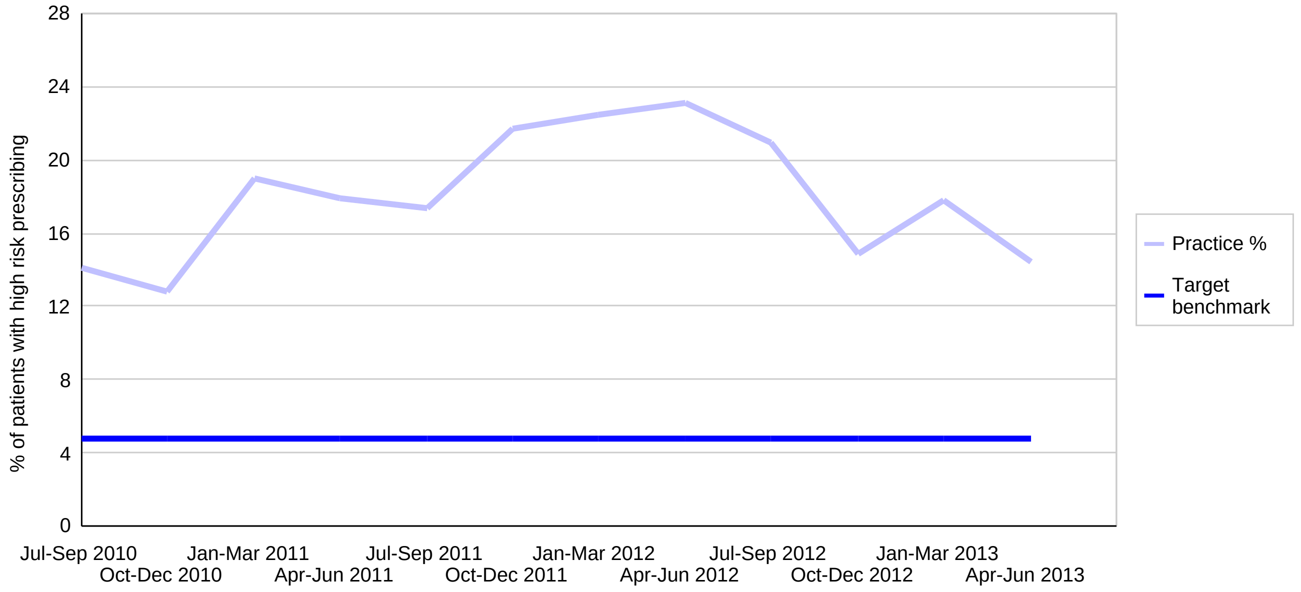

What does this indicator measure?

This indicator measures the percentage of patients on renal toxic drugs who are prescribed an oral NSAID. Patients included are all prescribed a diuretic along with an ACE inhibitor or Angiotensin Receptor Blocker (ARB - e.g. losartan, irbesartan etc). Recent articles have coined the term 'triple whammy' to emphasise the high renal risk associated with patients prescribed all three of NSAIDs (including Cox-2 selective agents), diuretics and ACEI/ARB, especially if they become dehydrated due to other illnesses. The fatality rate for cases of acute renal failure with the "triple whammy" may be as high as 10%. The triple whammy combination should be avoided in all patients, but the risk is likely to be higher in elderly patients and particularly those with pre-existing renal impairment or with chronic heart failure. If the NSAID is judged to be essential, then the lowest effective dose should be used for the shortest possible duration. All patients will require monitoring of renal function and should be advised to seek medical advice if they encounter conditions, such as diarrhoea or vomiting, since dehydration in these patients may precipitate acute renal failure. The safest option is always to avoid prescribing the NSAID to patients already prescribed diuretics and ACEI/ARBs.

What does the information show for my practice?

You had 15 (14.42%) patients aged >=65 and taking a diuretic and an ACEI/ARB who were prescribed an NSAID in April to June 2013. This was higher than the benchmark of 4.76% (the dark blue line benchmark is set at the percentage achieved by the quarter of practices with the lowest rates in financial year 2011/12). These prescriptions are high risk and we encourage you to review these patients to ensure that the prescription is appropriate. Searches to identify these patients in Vision or EMIS are available at www.isdscotland.org/efipps33.

Indicator 3. Older person (>=75 years) prescribed an NSAID without gastroprotection

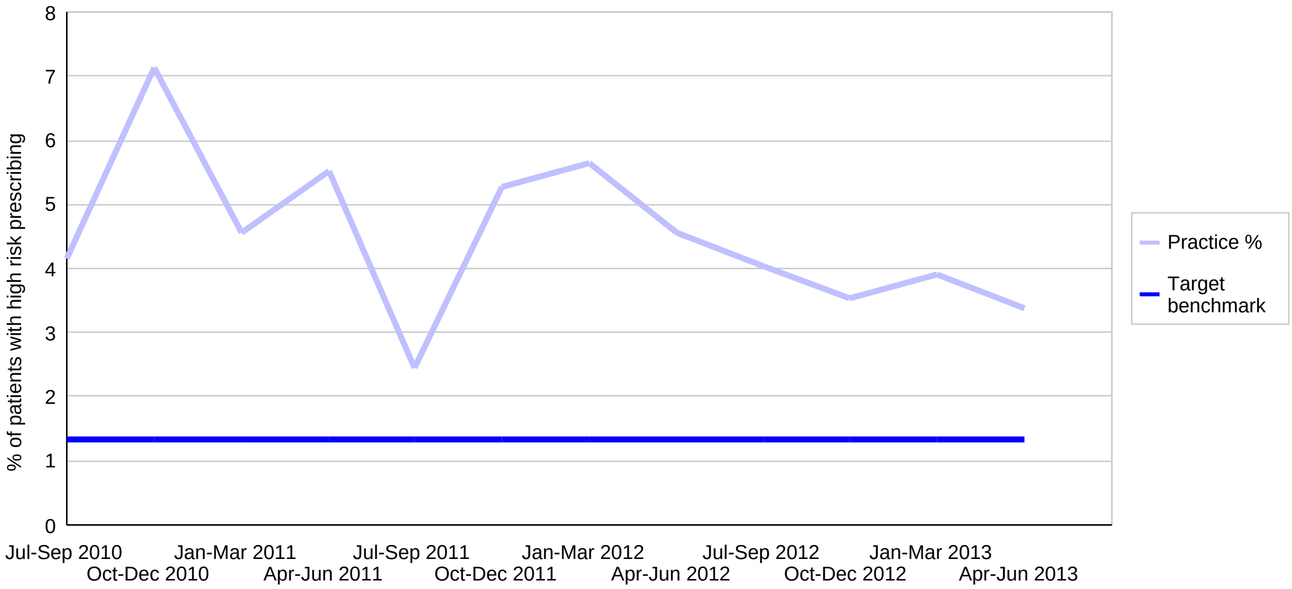

What does this indicator measure?

This indicator measures the percentage of patients aged 75 and over who are prescribed an NSAID without gastro-protection.

Why does this matter?

Oral NSAIDs increase the risk of gastrointestinal bleeding at all ages, but people aged >65 years have 5 to 6 times the risk of younger people, and those aged >75 years have 10 times the risk. As well as the risk of GI bleeding, older people will often have other reasons to avoid NSAIDS, including ischaemic heart disease, heart failure, renal impairment, and interactions with other drugs. In the absence of clear inflammation, NSAIDs have no clear analgesic benefit over other drugs, although individual response to painkillers varies. NICE advises that for osteoarthritis and minor musculoskeletal injury in the elderly, *full-dose* paracetamol and/or *topical* NSAIDs should be tried first and will be effective in the majority of patients. If other analgesics have been tried and oral NSAIDs/ Cox-2 selective inhibitors are judged necessary, these agents should be used at the lowest effective dose for the shortest possible period of time. Standard-dose ibuprofen (<=1200mg per day) is recommended as an appropriate first choice NSAID in view of its relatively lower risk of gastrointestinal (GI) and cardiovascular (CV) side effects. Current guidance also states that all older patients prescribed an oral NSAID should be co-prescribed gastroprotection, using a proton pump inhibitor as first choice. This reduces the excess risk of GI bleeding, but does not abolish it, and the safest course of action is always to avoid the NSAID if possible.

What does the information show for my practice?

You had 8 (3.39%) patients aged >=75 who were prescribed an NSAID without gastroprotection in April to June 2013. This was higher than the benchmark of 1.32% (the dark blue line benchmark is set at the percentage achieved by the quarter of practices with the lowest rates in financial year 2011/12). These prescriptions are high risk and we encourage you to review these patients to ensure that the prescription is appropriate. Searches to identify these patients in Vision or EMIS are available at www.isdscotland.org/efipps33.

Indicator 4. Older person (>=65 years) currently taking either aspirin or clopidogrel who is prescribed an NSAID without gastroprotection

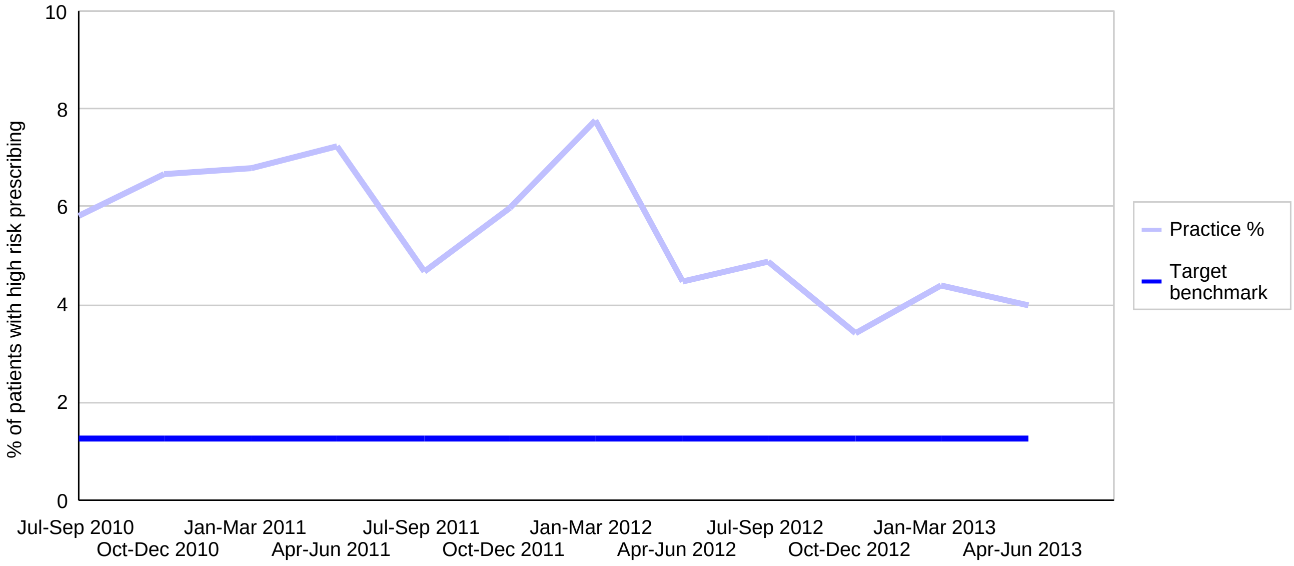

What does this indicator measure?

This indicator measures the percentage of patients aged 65 and over who take aspirin or clopidogrel, and who are also prescribed an oral NSAID.

Why does this matter?

Aspirin alone approximately doubles the risk of GI bleeding and co-prescribing an NSAID increases that risk to approximately 8-fold compared to untreated patients. Co-prescription of an NSAID with clopidogrel carries similar risks. The Committee for the Safety of Medicines (CSM) has advised that co-prescription of low dose aspirin and NSAIDs should only be used if absolutely necessary. If combined treatment with low dose aspirin and NSAIDs is considered to be essential, then using a PPI like omeprazole or lansoprazole reduce GI risk is superior to replacing traditional NSAIDs by Cox-2 selective agents (without gastro-protection), because low dose aspirin negates the gastro-protective effect of Cox-2 selective NSAIDs. In addition, ibuprofen and naproxen (but NOT diclofenac) have a more favourable cardiovascular risk profile than Cox-2 selective NSAIDs and therefore have advantages in patients using low dose aspirin for cardio-protection. Gastro-protection with a PPI reduces the excess risk of GI bleeding, but does not abolish it, and the safest course of action is always to avoid the NSAID if possible.

What does the information show for my practice?

You had 9 (4.00%) patients aged >=65 and taking aspirin or clopidogrel who were prescribed an NSAID without gastroprotection in April to June 2013. This was higher than the benchmark of 1.25% (the dark blue line benchmark is set at the percentage achieved by the quarter of practices with the lowest rates in financial year 2011/12). These prescriptions are high risk and we encourage you to review these patients to ensure that the prescription is appropriate. Searches to identify these patients in Vision or EMIS are available at www.isdscotland.org/efipps33.

Indicator 5. Current oral anticoagulant user prescribed an NSAID without gastroprotection

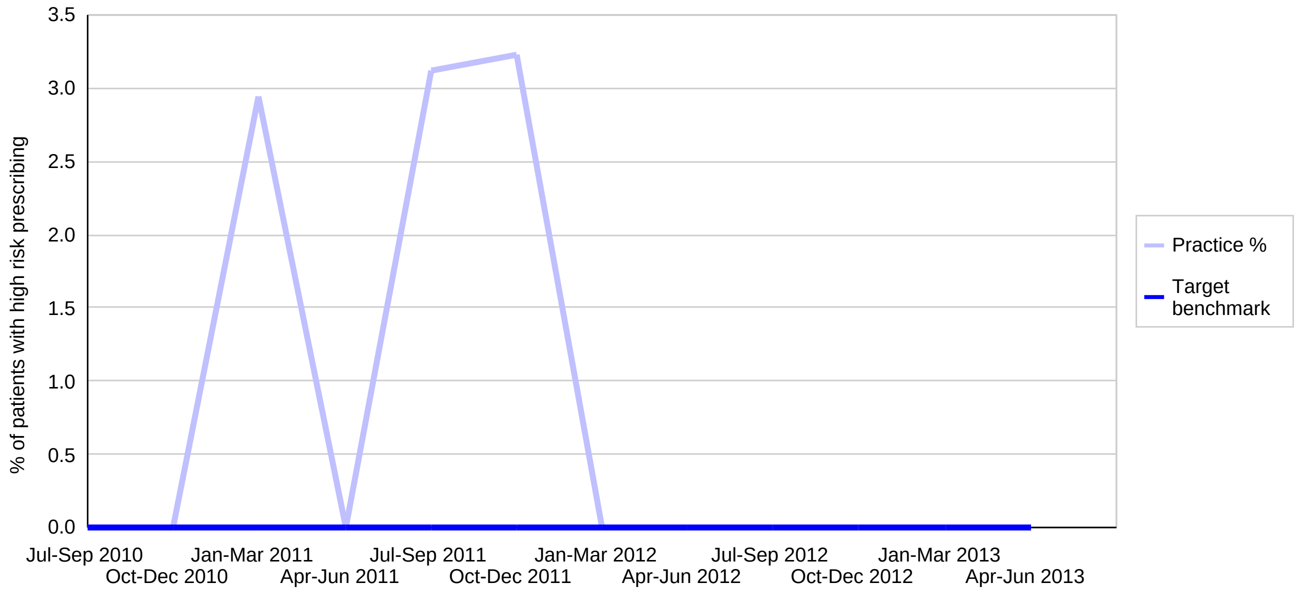

What does this indicator measure?

The indicator measures whether patients prescribed an oral anticoagulant (usually warfarin) in each quarter were also prescribed an oral NSAID (creams and gels are ignored).

Why does this matter?

The co-prescription of oral anticoagulants and oral NSAIDs is high risk because oral NSAIDs increase the risk of gastrointestinal bleeding, which is more dangerous if the patient is anti-coagulated. Warfarin alone increases the risk of gastro-intestinal bleeding approximately two-fold, and co-prescription of an NSAID increases that risk to 3 to 8-fold compared to untreated patients. Oral NSAIDs may also directly enhance the anti-coagulant effect of warfarin, although this is probably less important. If NSAID prescribing is *essential* (which it will occasionally be), then additionally prescribing a gastroprotective drug partially reduces the increased risk of GI bleeding caused by the NSAID, but it does not abolish it, and PPIs themselves (omeprazole etc) may enhance the anti-coagulant effect of warfarin. However, the safest thing to do is to stop the NSAID and avoid it in the future.

What does the information show for my practice?

You had no patients taking warfarin who were prescribed an NSAID without gastroprotection in April to June 2013.

NB - your practice had 40 patients taking warfarin, so you are only likely to have small numbers of these patients prescribed an NSAID without gastroprotection at any time. Your line on the graph may therefore be quite unstable. However, it is still important to avoid this prescribing if possible, and review it if it occurs.

Indicator 6. Current oral anticoagulant user prescribed aspirin or clopidogrel without gastroprotection

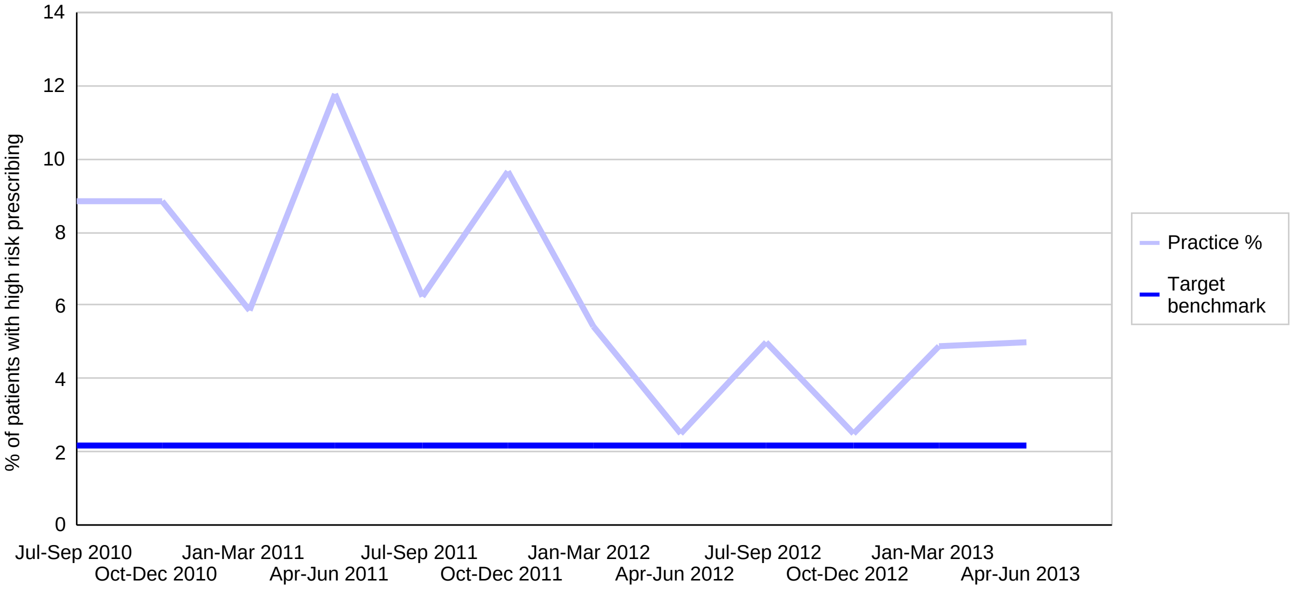

What does this indicator measure?

The indicator measures whether patients prescribed an oral anticoagulant (usually warfarin) in each quarter were also prescribed aspirin or clopidogrel.

Why does this matter?

The co-prescription of oral anticoagulants and aspirin or clopidogrel is high risk because aspirin and clopidogrel increase the risk of gastrointestinal bleeding, which is more dangerous if the patient is anti-coagulated. Warfarin alone increases the risk of gastro-intestinal bleeding approximately two-fold and co-prescription of low dose aspirin increases that risk to 4 to 10-fold compared to untreated patients. Current guidelines state that dual treatment with antiplatelets and anticoagulants is indicated only for the management of some patients with prosthetic heart valves, for some people with peripheral arterial disease and failing grafts, and for up to 12 months in patients with atrial fibrillation after acute coronary syndrome including myocardial infarction (the latter is the most common indication). If the combination is not strongly indicated, then stopping the antiplatelet is advised, although you may wish to seek specialist advice if the indication is ambiguous. If combination prescribing is *essential*, then additionally prescribing a gastroprotective drug partially reduces the increased risk of GI bleeding caused by the combination, but it does not abolish it. PPIs themselves (omeprazole etc) may enhance the anti-coagulant effect of warfarin, so increased monitoring at the time of initiation is indicated. Current guidance also recommends NOT using omeprazole or esomeprazole in patients prescribed clopidogrel, and using an alternative PPI instead.

What does the information show for my practice?

You had 2 (5.00%) patients taking warfarin who were prescribed aspirin or clopidogrel without gastroprotection in April to June 2013. This was higher than the benchmark of 2.19% (the dark blue line benchmark is set at the percentage achieved by the quarter of practices with the lowest rates in financial year 2011/12). These prescriptions are high risk and we encourage you to review these patients to ensure that the prescription is appropriate. Searches to identify these patients in Vision or EMIS are available at www.isdscotland.org/efipps33.

NB - your practice had 40 patients taking warfarin, so you are only likely to have small numbers of these patients prescribed aspirin or clopidogrel without gastroprotection at any time. Your line on the graph may therefore be quite unstable. However, it is still important to avoid this prescribing if possible, and review it if it occurs.
